# Unconventional T Cells in IgA Nephropathy

**DOI:** 10.1016/j.ekir.2024.12.017

**Published:** 2024-12-20

**Authors:** Zizhang Sheng, Nicholas J. Steers

**Affiliations:** 1 Aaron Diamond AIDS Research Center, Columbia University Vagelos College of Physicians and Surgeons, New York, New York, USA; 2 Division of Infectious Diseases, Department of Medicine, Columbia University Vagelos College of Physicians and Surgeons, New York, New York, USA; 3 Division of Nephrology, Department of Medicine, Columbia University Irving Medical Center, New York, New York, USA

See Clinical Research on Page 475

IgA nephropathy (IgAN) is the most common form of glomerulonephritis, and it is estimated that 20% to 40% of patients will develop end-stage renal disease within 20 years of the initial diagnosis.^1^ IgAN is characterized by the detection of IgA immune complexes containing galactose-deficient IgA1 (Gd-IgA1), deposited in the kidney mesangium observed in kidney biopsies. Although not diagnostic, patients with IgAN typically have elevated levels of IgA1 and Gd-IgA1 in the circulation compared to healthy individuals.^2^

There is a multihit model of IgAN based on previous genetic and immunological data that has been proposed.^S1–S3^ The initial hit is the generation of Gd-IgA1 followed by anti-Gd-IgA1 antibodies (hit 2) and the generation of Gd-IgA1 immune complexes (hit 3), and the final hit is the deposition of immune complexes in the kidney mesangium, causing kidney inflammation, release of proinflammatory cytokines, and the activation of the complement system. The immune system plays an important role in the pathogenesis of IgAN; however, the molecular basis of immune modulation of IgAN pathogenesis is poorly understood.

Previous research over the last 4 decades has demonstrated that mucosal infections and abnormalities in the mucosal immune response are associated with IgAN activation, and some of the current therapeutics are specifically targeting the mucosal immune response.^3^ Supporting the evidence of the dysregulated mucosal immune response, the intestinal microbiome can post translationally modify IgA1 molecules.^4^ Typically, monomeric IgA1 in the circulation is generated in the bone marrow and in much smaller quantities compared with multimeric IgA1 found in lamina propria of mucosal tissues. One of the proposed mechanisms for the misplaced mucosal multimeric IgA1 in the circulation of patients with IgAN is that mucosal B cells relocate to other tissues to produce and secrete mucosal multimeric IgA1 into the circulation, forming immune complexes that are deposited in the kidney glomerulus. Observations supporting this include that multimeric IgA1 found in the circulation of patients with IgAN has an *O*-glycosylation pattern that is characteristic of mucosal IgA1^5^ and circulating T helper cells overexpress cell surface receptors directing cells to the bone marrow.^6^ However, these are a series of observations, and additional studies need to be conducted to confirm and elucidate the mechanisms of immune cells leaving mucosal tissues, entering the circulation, and taking up residence in nonmucosal tissues such as the bone marrow and the kidney.

Currently, no good IgAN mouse models are available, because mice do not generate an equivalent IgA1 molecule. In humans, the immunological studies of IgAN primarily focuses on the peripheral blood mononuclear cells and not tissue resident cells. With the development of methods, including spectral flow cytometry and CyTOF to analyze peripheral blood mononuclear cells, it is possible to analyze multiple cell populations in the circulation in a single sample. This allows one to phenotypically characterize the rare populations associated with IgAN in the circulation, including cells that should reside in mucosal tissues and either not be present or present in low numbers in the circulation. One of these cells is the unconventional T cells which are poorly defined and cannot be classified into the “helper”, “cytotoxic”, and “regulatory” T cell populations; these cells are typically not restricted by the classical major histocompatibility complex molecules. Examples of these unconventional T cells include the mucosal associated invariant T cells (MAIT), γδ T cells, and natural killer T cells,^7^^,^^8^ and all are capable of activating B cells and class-switching naïve B cells into memory cells and plasma cells. Unconventional T cells and in particular MAIT cells have roles in autoimmunity and suppressing the immune response^S4^; thus, it is essential to understand these cells in more detail in IgAN.^8^

Gentile *et al.*,^9^ used a CyTOF approach to analyze the circulating immune cells, including B cells, T cells, myeloid cells, and the unconventional T cells that typically reside in mucosal tissues and not in the circulation. This is one of the first studies that has analyzed the subsets of both the humoral and cellular immune cell populations in patients with IgAN compared with healthy individuals. Gentile *et al.*, initially described an increase in the double negative B cell populations and a reduction of the transitional B cell populations expressing gut homing markers in patients with IgAN compared with healthy individuals. The authors proposed the observed decrease in the transitional B cells, and these cells in patients with IgAN are preferentially located in the gut.

Gentile *et al.*,^9^ analyzed the conventional and unconventional T cell populations in patients with IgAN compared with healthy individuals. Their phenotypic analysis demonstrated that there were no phenotypic changes in the conventional CD4+ and CD8+ T cell populations; however, a significant decrease in the MAIT cell and the γδ T cell populations was observed in patients with IgAN compared with healthy individuals. The reduction of the MAIT cells and γδ T cells in the circulation may be because these cells are being recruited to mucosal tissues, thus promoting the generation of mucosal polymeric-IgA1. To the best of our knowledge, this one of the first studies to examine the immune cells simultaneously and to identify differences in the MAIT cell and the γδ T cell populations in the circulation of patients with IgAN compared with healthy controls. However, the exhausted unconventional T cell population was increased in patients with IgAN. The accumulation of exhausted unconventional T cells may indicate that these cells are being chronically stimulated, because this phenotype has been shown to accumulate following severe viral infections and in cancer. The exhausted unconventional T cells may also become less functionally active; however, the functional capacity of these cells in patients with IgAN and healthy individuals needs to be elucidated.

The study reveals alterations in B cell and unconventional T cell populations in the circulation of patients with IgAN compared with healthy individuals. However, it remains unknown how these populations are influenced during different states of IgAN, including disease activation and disease remission. Conventional T cells and unconventional T cells are important in the generation of the humoral immune response and IgA1 in mucosal tissues. There have been transcriptomic studies of the conventional T cell, specifically the T follicular–like helper cells in IgAN. The data presented in the study by Gentile *et al.*, demonstrated the importance of evaluating the unconventional T cells in IgAN, both phenotypically and functionally. Evaluating unconventional T cell populations longitudinally will determine how these populations are altered in different disease states and if there is an increase in the exhausted T cells during disease exacerbation. Transcriptional analysis of these unconventional T cells will generate data to begin to understand the functional properties of these cells, and the transcriptomic analysis will identify if there are unique signatures and clonal expansion of these cells. Understanding the role of unconventional T cells in IgAN and the molecular mechanisms of how unconventional T cells promote the generation of IgA1 and Gd-IgA1 in IgAN, may reveal novel therapeutic targets.

## Disclosure

NJS is on the Editorial Board of Kidney International Reports. ZS reports no competing interests.

## Supplementary Material

Supplementary File (PDF) **Supplementary References**.
