# Supplementary material for: Unconventional T Cells in IgA Nephropathy
Source: Kidney Int Rep. 2024 Dec 20;10(2):296–8. doi: 10.1016/j.ekir.2024.12.017 (PMC11843308; doi:10.1016/j.ekir.2024.12.017)
Supplement: Supplementary File (PDF) — Supplementary References. [file mmc1.pdf]

Supplementary Material:

Supplemental References:

S1 Suzuki H, Kiryluk K, Novak J, et al. The pathophysiology of IgA nephropathy. JAm Soc Nephrol 2011; 22: 1795-1803.

S2 Kiryluk K, Novak J, Gharavi AG. Pathogenesis of immunoglobulin A nephropathy: recent insight from genetic studies. Annu Rev Med 2013; 64: 339-356.

S3 Kiryluk K, Novak J. The genetics and immunobiology of IgA nephropathy. J Clin Invest 2014; 124: 2325-2332.

S4 Kaminski H, Couzi L, Eberl M. Unconventional T cells and kidney disease. Nat Rev Nephrol 2021; 17: 795-813.
